# Configuration and Specifications of an Unmanned Aerial Vehicle (UAV) for Early Site Specific Weed Management

**DOI:** 10.1371/journal.pone.0058210

**Published:** 2013-03-06

**Authors:** Jorge Torres-Sánchez, Francisca López-Granados, Ana Isabel De Castro, José Manuel Peña-Barragán

**Affiliations:** Department of Crop Protection, Institute for Sustainable Agriculture (IAS) Spanish National Research Council (CSIC), Córdoba, Spain; University of Adelaide, Australia

## Abstract

A new aerial platform has risen recently for image acquisition, the Unmanned Aerial Vehicle (UAV). This article describes the technical specifications and configuration of a UAV used to capture remote images for early season site- specific weed management (ESSWM). Image spatial and spectral properties required for weed seedling discrimination were also evaluated. Two different sensors, a still visible camera and a six-band multispectral camera, and three flight altitudes (30, 60 and 100 m) were tested over a naturally infested sunflower field. The main phases of the UAV workflow were the following: 1) mission planning, 2) UAV flight and image acquisition, and 3) image pre-processing. Three different aspects were needed to plan the route: flight area, camera specifications and UAV tasks. The pre-processing phase included the correct alignment of the six bands of the multispectral imagery and the orthorectification and mosaicking of the individual images captured in each flight. The image pixel size, area covered by each image and flight timing were very sensitive to flight altitude. At a lower altitude, the UAV captured images of finer spatial resolution, although the number of images needed to cover the whole field may be a limiting factor due to the energy required for a greater flight length and computational requirements for the further mosaicking process. Spectral differences between weeds, crop and bare soil were significant in the vegetation indices studied (Excess Green Index, Normalised Green-Red Difference Index and Normalised Difference Vegetation Index), mainly at a 30 m altitude. However, greater spectral separability was obtained between vegetation and bare soil with the index NDVI. These results suggest that an agreement among spectral and spatial resolutions is needed to optimise the flight mission according to every agronomical objective as affected by the size of the smaller object to be discriminated (weed plants or weed patches).

## Introduction

Precision agriculture (PA) is defined as “*a management strategy that uses information technology to bring data from multiple sources to bear on decisions associated with crop production*” [1]. PA encompasses all the techniques and methods for crop and field management by taking into account their local and site-specific heterogeneity and variability [2]. Within the context of PA, early season site-specific weed management (ESSWM) involves the development of techniques to detect the weeds growing in a crop and the application of new technologies embedded in specific agricultural machinery or equipment to control them successfully, taking action to maximise economic factors and reduce the environmental impact of the control measurements applied [3]. The efficient development of these practices somehow relies on the use of remote sensing technology for collecting and processing spatial data from sensors mounted in satellite or aerial platforms. This technology has been widely applied in agricultural studies, allowing the mapping of a variety of factors [4], including crop conditions [5], soil properties [6], water content [7] and weed distribution [8], among others. Piloted aircraft and satellites are traditionally the primary platforms used to obtain remote images for local to global data acquisition. However, these platforms present problems for many aspects of precision agriculture because they are limited in their ability to provide imagery of adequate spatial and temporal resolutions and are strongly affected by weather conditions [9]. In the case of ESSWM, good results have been obtained in late growth stages (normally at the flowering stage) using aerial [10]–[11] and satellite [12] images, with herbicide savings of more than 50% reported. Nevertheless, in most weed-crop scenarios, the optimal weed treatment is recommended at an early growth stage of the crop, just a few weeks after crop emergence. In this stage, mapping weeds using remote sensing presents much greater difficulties than in the case of the late-stage season for three main reasons [13]: 1) weeds are generally distributed in small patches, which makes it necessary to work with remote images at very small pixel sizes, often on the order of centimetres [14]; 2) grass weeds and monocotyledonous crops (e.g., *Avena* spp. in wheat) or broad-leaved weeds and many dicotyledonous crops (e.g., *Chenopodium* spp. in sunflower) generally have similar reflectance properties early in the season, which decreases the possibility of discriminating between vegetation classes using only spectral information; and 3) soil background reflectance may interfere with detection [15].

Today, difficulties related to spatial and temporal resolutions can be overcome using an Unmanned Aerial Vehicle (UAV) based remote sensing system, which has progressed in recent years as a new aerial platform for image acquisition. UAVs can fly at low altitudes, allowing them to take ultra-high spatial resolution imagery and to observe small individual plants and patches, which has not previously been possible [16]. Moreover, UAVs can supply images even on cloudy days, and the time needed to prepare and initiate the flight is reduced, which allows greater flexibility in scheduling the imagery acquisition. Other advantages of UAVs are their lower cost, and the lower probability of serious accidents compared with piloted aircraft.

Examples of applications of UAVs in agricultural studies are becoming more noticeable in the literature. For instance, Hunt *et al.* (2005) [17] evaluated an aerobatic model aircraft for acquiring high-resolution digital photography to be used in estimating the nutrient status of corn and crop biomass of corn, alfalfa, and soybeans. In other cases, an unmanned helicopter was tested to monitor turf grass glyphosate application [16], demonstrating its ability to obtain multispectral imaging. Other UAV models have been developed, such as the six-rotor aerial platform used by Primicerio *et al.* (2012) [18] to map vineyard vigour with a multi-spectral camera. Recently, Zhang and Kovacs (2012) [19] reviewed the advances in UAV platforms for PA applications. In this review, they indicated the phases in the production of the remote images (including acquisition, georeferencing and mosaicking) and the general workflow for information extraction. Generally, all these authors concluded that these systems provide very promising results for PA and identified some key factors for equipment and system selection, such as maximum UAV payload capacity, platform reliability and stability, sensor capability, flight length and UAV manoeuvrability, among others [20]–[22].

To our knowledge, however, no detailed investigation has been conducted regarding the application of this technology in the field of ESSWM, in which remote images at centimetre-scale spatial resolution and a narrow temporal window for image acquisition are required [23]. Therefore, this paper defines the technical specifications and configuration of a quadrocopter UAV and evaluates the spatial and spectral requirements of the images captured by two different sensors (a commercial scale camera and a multispectral 6-channel camera) with the ultimate aim of discriminating weed infestations in a sunflower crop-field in the early growing season for post-emergence treatments. Moreover, the steps for preparing and performing UAV flights with both cameras are described as well as the relationships amongst flight altitude, pixel size, sensor properties and image spectral information.

## Materials and Methods

### 1. UAV Description

**Figure 1:**
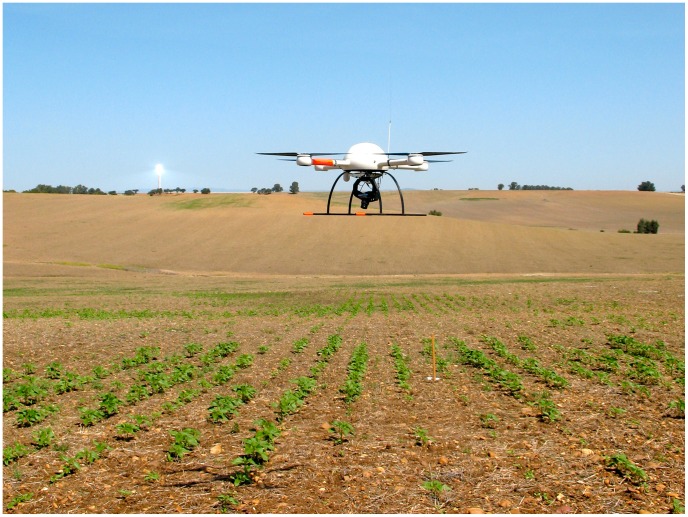
The quadrocopter UAV, model md4-1000, flying over the experimental crop-field.

A quadrocopter platform with vertical take-off and landing (VTOL), model md4-1000 (microdrones GmbH, Siegen, Germany), was used to collect a set of aerial images at several flight altitudes over an experimental crop-field (Figure 1). This UAV is equipped with four brushless motors powered by a battery and can fly by remote control or autonomously with the aid of its Global Position System (GPS) receiver and its waypoint navigation system. The VTOL system makes the UAV independent of a runway, so it can be used in a wide range of different situations and flight altitudes. The UAV’s technical specifications and operational conditions, provided by the manufacturer, are shown in Table 1.

**Table 1:** Technical specifications and operational conditions of the UAV, model md4-1000.

UAV specification	Value
*Technical specifications*	
Climb rate	7.5 m/s
Cruising speed	15.0 m/s
Peak thrust	118 N
Vehicle mass	2.65 Kg approx. (depends on configuration)
Recommended payload mass	0.80 Kg
Maximum payload mass	1.25 Kg
Maximum take-off weight	5.55 Kg
Dimensions	1.03 m between opposite rotor shafts
Flight time	Up to 45 min (depends on payload and wind)
*Operational conditions*	
Temperature	−10°C to 50°C
Humidity	Maximum 90%
Wind tolerance	Steady pictures up to 6 m/s
Flight radius	Minimum 500 m using radiocontrol, with waypoints up to 40 km
Ceiling altitude	Up to 1,000 m
Take-off altitude	Up to 4,000 m about sea level

Source: UAV manufacturer (microdrones GmbH, Siegen, Germany).

The whole system consists of the vehicle, the radio control transmitter, a ground station with the software for mission planning and flight control, and a telemetry system. The radio control transmitter is a handheld device whose main tasks are to start the vehicle’s engines, manage take-off and landing, control the complete flight in the manual mode, and activate the autonomous navigation system. The control switchboard consists of several triggers, pushbuttons, scroll bars, a display, and an antenna, and it is equipped with a RF-module synthesiser, which enables the selection of any channel in the 35 MHz band. The ground station works as an interface between the operator and the vehicle and includes the support software mdCockpit (MDC). MDC allow the UAV settings to be configured, implements the flight route plan with the Waypoint Editor (WPE) module, and monitors the flight. The telemetry system collects relevant flight data and retrieves a stream of information in a plain text scheme that includes GPS position data, attitude, altitude, flight time, battery level, and motor power output, among many others. All sensors and control devices for flight and navigation purposes are embedded on-board the vehicle and are managed by a computer system, which can listen telemetry data and make decisions according to the momentary flight situation and machine status, thus avoiding that occasional loss of critical communication between the UAV and the ground station resulting in the vehicle crashing.

Three persons were employed for the secure use of the UAV: a radio control pilot, a ground station operator and a visual observer. The radio control pilot manually takes off and lands the UAV and activates the programmed route during the flight operation. The ground station operator controls the information provided by the telemetry system, i.e., UAV position, flight altitude, flight speed, battery level, radio control signal quality and wind speed. The visual observer is on the lookout for potential collision threats with other air traffic.

### 2. Sensors Description

The md4-1000 UAV can carry any sensor weighing less than 1.25 kg mounted under its belly, although the maximum recommended payload is 0.80 kg. Two sensors with different spectral and spatial resolutions were separately mounted on the UAV to be tested in this experiment: a still point-and-shoot camera, model Olympus PEN E-PM1 (Olympus Corporation, Tokyo, Japan), and a six-band multispectral camera, model Tetracam mini-MCA-6 (Tetracam Inc., Chatsworth, CA, USA). The Olympus camera acquires 12-megapixel images in true colour (Red, R; Green, G; and Blue, B, bands) with 8-bit radiometric resolution and is equipped with a 14–42 mm zoom lens. The camera’s sensor is 4,032×3,024 pixels, and the images are stored in a secure digital SD-card. The mini-MCA-6 is a lightweight (700 g) multispectral sensor composed of six individual digital channels arranged in a 2×3 array. The slave channels are labelled from “1” to “5”, while the sixth “master” channel is used to define the global settings used by the camera (e.g., integration time). Each channel has a focal length of 9.6 mm and a 1.3 megapixel (1,280×1,024 pixels) CMOS sensor that stores the images on a compact flash CF-card. The images can be acquired with 8-bit or 10-bit radiometric resolution. The camera has user configurable band pass filters (Andover Corporation, Salem, NH, USA) of 10-nm full-width at half-maximum and centre wavelengths at B (450 nm), G (530 nm), R (670 and 700 nm), R edge (740 nm) and near-infrared (NIR, 780 nm). These bandwidth filters were selected across the visible and NIR regions with regard to well-known biophysical indices developed for vegetation monitoring [24]. Image triggering is activated by the UAV according to the programmed flight route. At the moment of each shoot, the on-board computer system records a timestamp, the GPS location, the flight altitude, and vehicle principal axes (pitch, roll and heading).

### 3. Study Site and Field Sampling

The UAV system was tested in a sunflower field situated at the private farm La Monclova, in La Luisiana (Seville, southern Spain, coordinates 37.527N, 5.302W, datum WGS84). The flights were authorized by a written agreement between the farm owners and our research group. We selected sunflower because this is the major oil-seed crop grown in Spain, with a total surface of 850,000 ha in 2012 [25], and because weed control operations (either chemical or physical) with large agricultural machinery represent a significant proportion of production costs, create various agronomic problems (soil compaction and erosion) and represent a risk for environmental pollution. The sunflower seeds were planted at the end of March 2012 at 6 kg ha^−1^ in rows 0.7 m apart. The set of aerial images were collected on May 15^th^, just when post-emergence herbicide or other control techniques are recommended in this crop. Several visits were periodically made to the field from crop sowing to monitor crop growth and weed emergence and, finally, to select the best moment to take the set of remote images. The sunflower was at the stage of 4–6 leaves unfolded. The weed plants had a similar size or, in some cases, were smaller than the crop plants (Figure 1).

**Figure 2:**
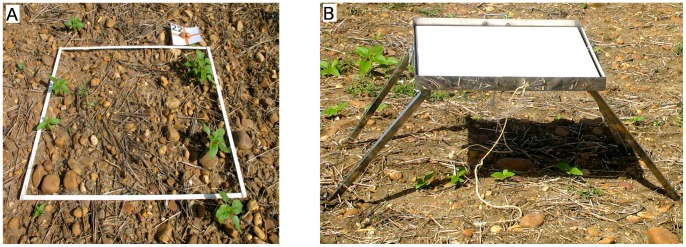
Details of the experimental set. a) 1×1 m frame used in the ground-truth field sampling, and b) reference panel for image spectral calibration.

An experimental plot of 100×100 m was delimited within the crop-field to perform the flights. The coordinates of each corner of the flight area were collected using GPS to prepare the flight route in the mission-planning task. A systematic on-ground sampling procedure was carried out the day of the UAV flights. The procedure consisted of placing 49 square white frames of 1×1 m distributed regularly throughout the studied surface (Figure 2A). Every frame was georeferenced with a GPS and photographed in order to compare on-ground weed infestation (observed weed density) and outputs from image classification (estimated weed density). These numbered cards were also utilised as artificial terrestrial targets (ATTs) to perform the imagery orthorectification and mosaicking process. In the course of the UAV flights, a barium sulphate standard spectralon® panel (Labsphere Inc., North Sutton, NH, USA) of 1×1 m was also placed in the middle of the field to calibrate the spectral data (Figure 2B).

### 4. UAV Flight and Sensors Tests

#### 4.1. Mission planning

**Figure 3:**
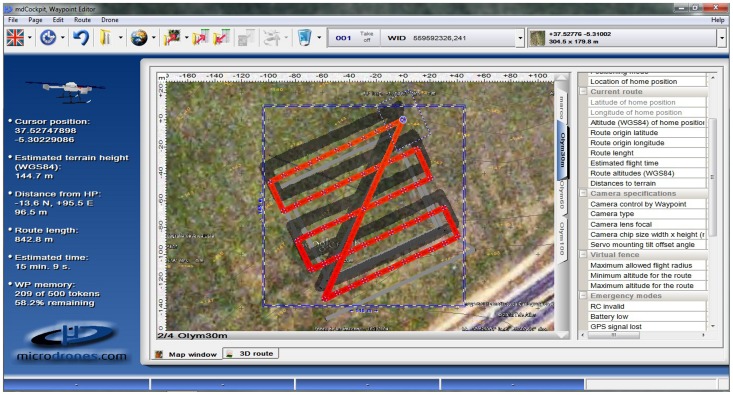
Screen shot of the Waypoint Editor module showing the flight planning.

The flight mission was planned with the WPE module of the MDC software installed at the ground station. The flight route was designed over the orthoimages and the digital elevation model (DEM) of the flight area previously imported from the application Google Earth™ (Keyhole Inc., Mountain View, CA, USA). Three different parameters were needed to plan the route: flight area, camera specifications and UAV tasks (Table 2). The flight area information includes width and length, the direction angle of the main side, and the desired overlap in the imagery. The images were acquired at 60% forward-lap and 30% side-lap. The camera specifications are the focal length and the sensor size. The UAV tasks refer to the actions that the UAV has to perform once it arrives at each point for image acquisition, and it includes the number of photos and dwell time in each point. Once both, this information and the flight altitude were introduced in the WPE module, it automatically generated the flight route and estimated the flight duration according to the total number of images planned (Figure 3). The route file was exported to a memory card embedded in the UAV via a standard serial link.

**Table 2:** Data required by the Waypoint Editor software and the route settings used in the experimental field.

Data type	Setting value*
*Flight area*	
Width	100 m
Length	100 m
Direction angle	65°
Horizontal overlapping	60%
Vertical overlapping	30%
*Camera specifications*	
Focal length	
RGB camera	14 mm
Multispectral camera	9.6 mm
Sensor size (width×length)	
RGB camera	17.3×13 mm
Multispectral camera	6.66×5.32 mm
*UAV tasks*	
Dwell	5 s
Number of images	1

* Values used in the experimental field.

#### 4.2. UAV flight and image acquisition

**Figure 4:**
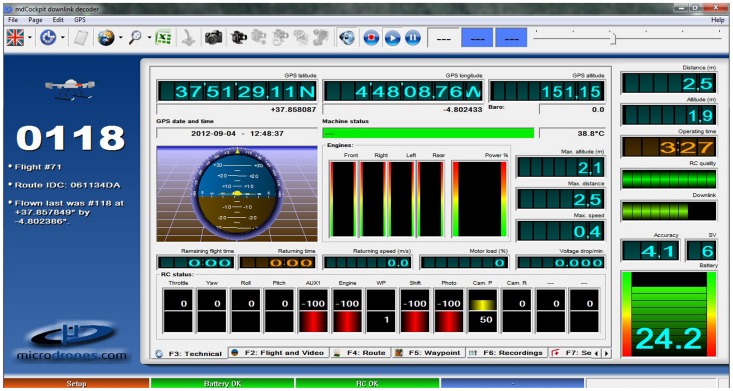
Screen shot of the Downlink Decoder module showing the information displayed during a programmed flight.

The preliminary steps before starting the flight were to upload the flight route to the UAV computer system, attach the camera to the vehicle and check the connectivity and the proper functioning of the whole system. After these steps, the pilot manually launches the UAV with the radio control transmitter and next activates the automatic flight route, making the vehicle go to the first waypoint and then fly along the flight lines until the entire study area is completely covered. Once all the images are taken, the pilot manually lands the UAV, and the ground station operator prepares the vehicle for the next route. During the flight, the ground station operator watches the UAV telemetry data using the downlink decoder, another component of the MDC software (Figure 4). This program gives information about: 1) operating time of the UAV, 2) current flight time, 3) distance from take-off point to the UAV, 4) quality of the remote control signal received by the UAV, 5) downlink quality, 6) battery status, and 7) GPS accuracy.

In addition to this information, the downlink decoder supports several important dialog pages, as follows:

Flight and video. This page shows the video stream captured by the sensor attached to the UAV, making it easier to control the UAV when it is manually driven. Additional data displayed in this page are: 1) distance to the UAV, 2) flight altitude above the take-off position, 3) speed of the UAV, 4) artificial horizon, 5) compass, and 6) roll and tilt angles.
Technical. This page supplies information about: 1) UAV position (GPS latitude and longitude), 2) UAV altitude (GPS altitude above sea level), 3) current navigation mode, 4) magnetometer status, 5) barometer status, 6) motor power, 7) momentary status of all the radio control channels, and 8) limit values of flight altitude, distance and speed.
Route. This page shows a tridimensional display of the flight path.
Waypoint. This section shows information about: 1) the flying route followed by the UAV, 2) the UAV GPS position, and 3) the waypoint command that is being executed at each moment.
Sensor-payload. This page displays a diagram with sensor data received from the payload.
Recordings. Three diagrams are displayed in this section: 1) comprising motor power and battery voltage over time, 2) comprising flight attitude (roll, pitch and yaw angles) with GPS data, and 3) comprising velocity, distance, wind profile, flight altitude and radio-control link quality.

#### 4.3. Multispectral band alignment

The images taken by the still camera (Olympus model) can be used directly after downloading to the computer, but those taken by the multispectral camera (mini-MCA-6 Tetracam model) require some pre-processing. This camera takes the images of each channel in raw format and stores them separately on six individual CF cards embedded in the camera. Therefore, an alignment process is needed to group the six images taken in each waypoint. The Tetracam PixelWrench 2 (PW2) software (Tetracam Inc., Chatsworth, CA, USA), supplied with the multispectral camera, was used to perform the alignment process. The PW2 software provides a band-to-band registration file that contains information about the translation, rotation and scaling between the master and slave channels. Two different options were tested: 1) basic configuration of the PW2 software, as applied by Laliberte *et al.* (2011) [26], and 2) advanced configuration of PW2, which includes the newest field of view (FOV) optical calculator, which calculates additional offsets to compensate the alignment for closer distances [27]. The quality of the alignment process was evaluated with the help of the spectralon® panel data captured in the images at a 30 m altitude. Spatial profiles were taken across the reference panel for each method and compared with the non-aligned image. The spatial profiles consisted of graphics representing the spectral values for each band along a line 45 pixels long drawn in the multi-band images using the ENVI image processing software (Research System Inc., Boulder, CO, USA).

#### 4.4. Spatial resolution and flight length as affected by flight altitude

Three independent flight routes were programmed for each type of camera to cover the whole experimental field at 30, 60 and 100 m altitude above ground level. The effects of flight altitude and camera resolution with respect to pixel size, area coverage (number of images per hectare) and flight duration were studied, and their implications for weed discrimination in the early season were discussed.

#### 4.5. Spectral resolution as affected by flight altitude

To perform weed mapping based on UAV images, two consecutive phases are usually required [13]: 1) bare soil and vegetation discrimination, which would allow obtaining a two-classes image with vegetal cover (crop and weeds together) and bare soil, 2) crop and weeds discrimination, in which the zones corresponding to crop are identified and masked, and finally, the detection and location of weeds are obtained. To determine the limitations of each sensor with regard to both phases, spectral values of the three covers present in the field (bare soil, crop and weeds) were extracted. These spectral values were collected in 15 randomly selected sampling areas for each soil use from the images taken during all the flight missions (i.e., both sensors at 30, 60 and 100 m altitudes).

Three well-known vegetation indices (VIs) were derived from these values:

Normalised Difference Vegetation Index (NDVI, [28])

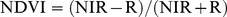
(1)- Normalised Green-Red Difference Index (NGRDI, [29]),



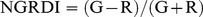
(2)- Excess Green Index (ExG, [30], [31]).



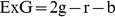
(3)The potential of the VIs for spectral discrimination was evaluated by performing a least significant difference (LSD) test at p≤0.01 through a one-way analysis of variance (ANOVA), and applying the M-statistic (equation 4) presented by Kaufman and Remer (1994) [32] in order to quantify the histograms separation of vegetation indices. JMP software (SAS, Cary, NC, USA) was employed to perform the statistical analysis.
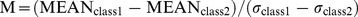
(4)


*M* expresses the difference in the means of the class 1 and class 2 histograms normalized by the sum of their standard deviations (σ). Following the research strategy and steps mentioned before, class 1 and class 2 were either, vegetation and bare soil, where vegetation was weeds and crop studied together, or weeds and crop. *M* values are indicative of the separability or discriminatory power of classes 1 and 2 considered in every step. Two classes exhibit moderate separability when *M* exceeds 1, showing easier separation for larger *M* values which will provide a reasonable discrimination [33]. According to Kaufman and Remer (1994) [32], the same difference in means can give different measures of separability depending on the spread of the histograms. Wider histograms (larger σ) will cause more overlap and less separability than narrow histograms (smaller σ) for the same difference in means.

## Results and Discussion

### 1. Image Pre-processing

#### 1.1. Band alignment of multispectral imagery

**Figure 5:**
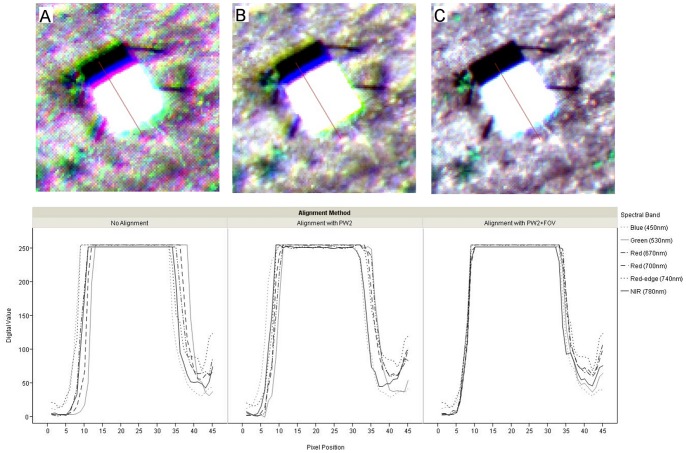
Images captured by the multispectral camera and spatial profiles depicting comparison of band-to-band alignment. a) No alignment, b) Alignment by using the basic configuration of the PW2 software, and c) Alignment by using the PW2 software plus the field of view (FOV) optical calculator.

The images acquired by both cameras were downloaded to a computer by inserting their memory cards into a card reader and copying the data. An alignment process was performed on the multispectral images to match the six bands into a single readable file. The alignment results were examined visually and evaluated using spatial profiles (Figure 5).

The displacement among the curves for each channel in the spatial profiles makes evident the band misalignment of the original non-aligned images. The non-aligned images showed halos around the reference objects (Spectralon and vegetation) and noise in the soil background (Figure 5A). These halos and noise were still recognisable in the image aligned using the basic configuration of the PW2 software (Figure 5B), although they were lesser than in the non-aligned image. These results are similar to those obtained by Laliberte *et al.* (2011) [26], who reported poor alignment results using PW2 software with the mini-MCA imagery. To solve this problem, they developed the local weighted mean transform (LMWT) method and obtained a satisfactory alignment. However, the latest version of the PW2 software, launched in 2012, which includes the FOV optical calculator, performed a good alignment and allowed elimination of the halos and a high reduction of the background noise (Figure 5C). In fact, these results seem to be quite similar to those achieved using the LMWT method. A good alignment of all the individual bands is crucial for subsequent image analysis, especially when spectral values of different objects of the image are extracted. The vegetation objects present in a weed-crop scenario in the early season are very small, as a consequence a poor alignment might include pixels not belonging to the objects of interest, drastically reducing the success of the image analysis and classification.

Next to the alignment process, the PW2 software generated a unique multi-band image file that is incompatible with the mosaicking software. Therefore, the last step was to convert this multi-band file to a TIFF-readable format using the ENVI software.

#### 1.2. Image orthorectification and mosaicking

**Figure 6:**
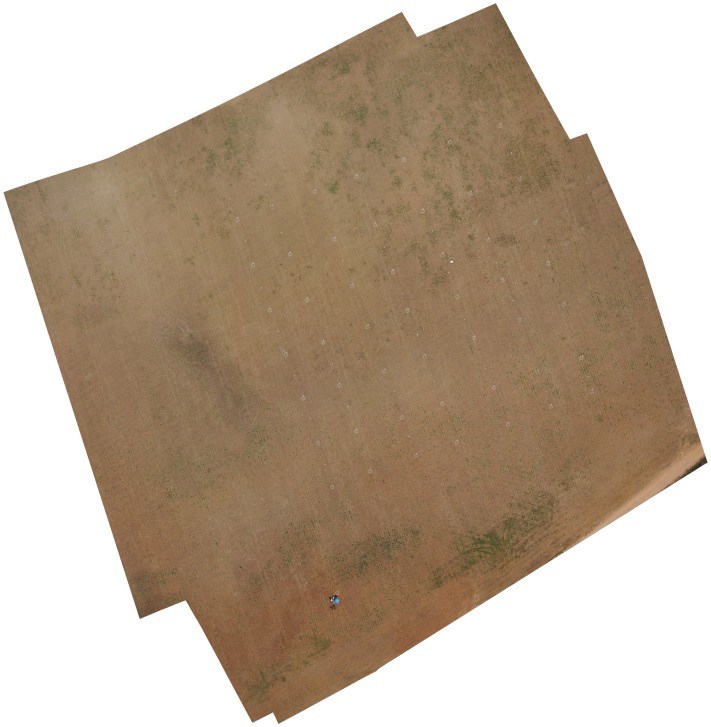
Ortho-mosaic of the whole experimental field. Composed from six individual images taken by the still RGB camera at 100 meters altitude.

A sequence of images was collected in each flight mission to cover the whole experimental crop-field. An important task prior to image analysis was the combination of all these individual and overlapped images by applying two consecutive processes of orthorectification and mosaicking. The Agisoft Photoscan Professional Edition (Agisoft LLC, St. Petersburg, Russia) software was employed in this task. In the first step, the software asks for the geographical position and principal axes (roll, pitch and yaw) of the vehicle in each acquired image. Next, the software automatically aligns the photos. Finally, some ATT’s coordinates are added to assign geographical coordinates to the image. Then, the software automatically performs the orthorectification and mosaicking of the imagery set into a single image of the whole experimental field (Figure 6). The resultant ortho-mosaic shows a high-quality landscape metric and accurate crop row matching between consecutive images, which guarantees good performance of the subsequent image classification.

### 2. Effect of Flight Altitude on Image Spatial Resolution and Flight Time

**Figure 7:**
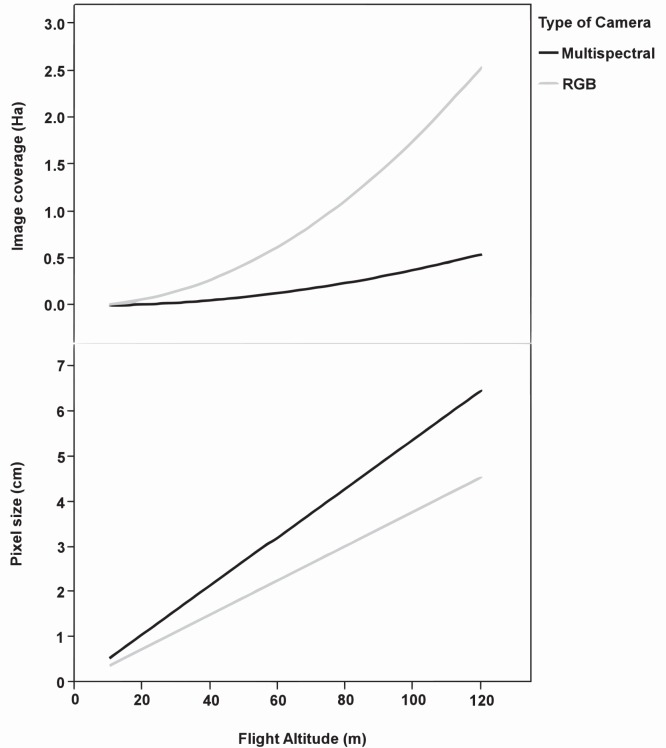
Image spatial resolution and coverage as affected by flight altitude and type of camera.

**Figure 8:**
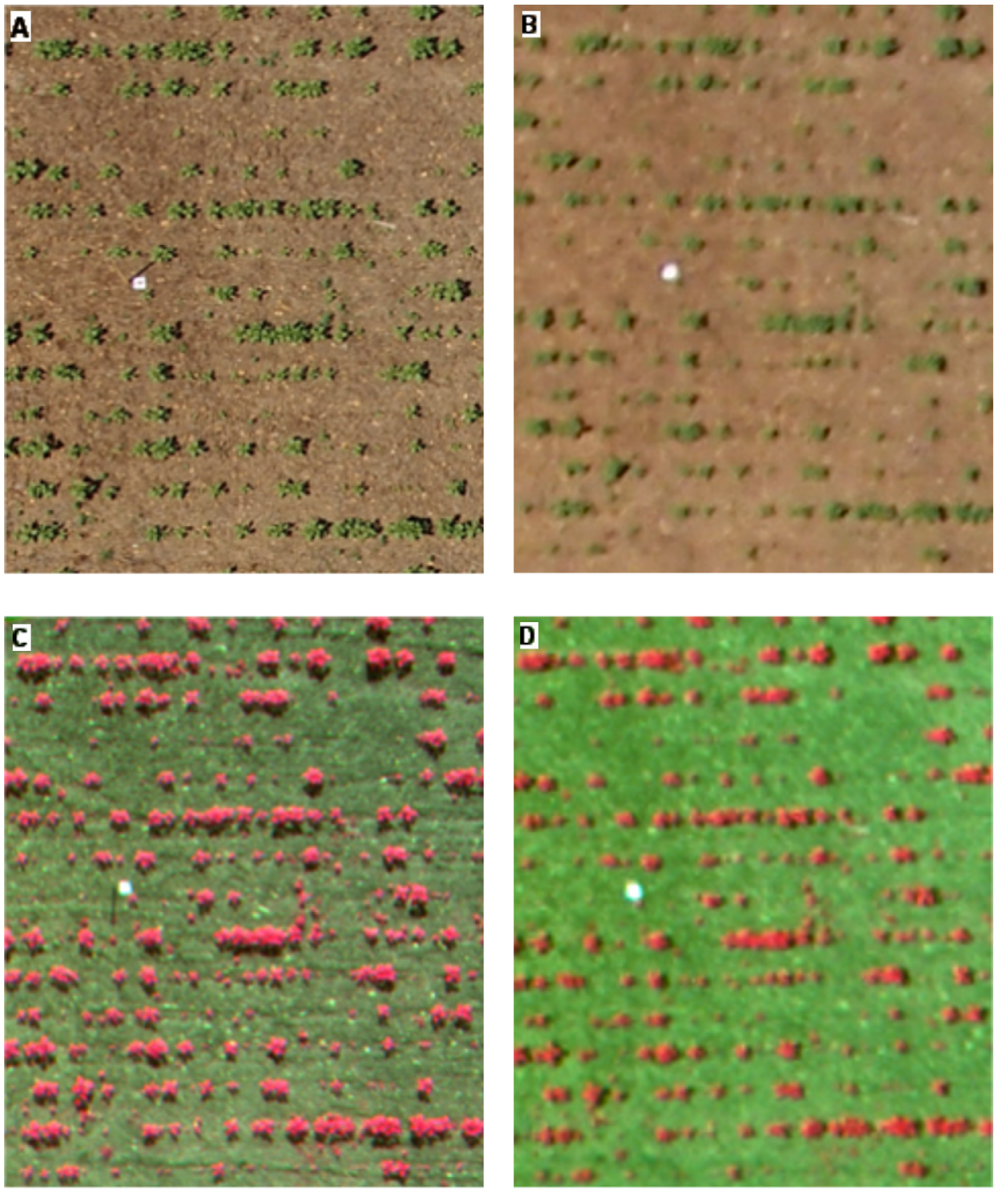
UAV images collected by the two cameras. Still RGB camera (a, b) and multispectral camera (c, d) at 30 m (a, c) and 100 m (b, d) flight altitude.

The image spatial resolution and the area covered by each image as affected by the UAV flight altitude and the type of camera are shown in Figure 7. The imagery pixel size was directly proportional to the flight altitude. The still RGB camera captured images with pixel sizes of 1.14 cm and 3.81 cm, while the multispectral camera captured images with pixel sizes of 1.63 cm and 5.42 cm at flight altitudes of 30 and 100 m, respectively (Figure 8). At these altitudes, the area covered by each image of the still RGB camera increased from 0.16 ha (46×35 m) to 1.76 ha (153×115 m) and of the multispectral camera from 0.04 (21×17 m) to 0.38 ha (69×55 m), respectively. The differences between both types of images were due to the cameras’ technical specifications (Table 2). The camera focal length affects both the pixel size and the area covered by each image, while the camera sensor size only influences the imagery pixel size.

A crucial feature of the remote images for weed mapping in the early season is their high spatial resolution, which can be achieved with low-altitude flights. Of great importance is defining the optimum pixel size needed according to each specific objective, which is calculated from the size of the weed seedlings to be discriminated, the distance between crop rows and the crop type. In general, at least four pixels are required to detect the smallest objects within an image [34]. Accordingly, if the objective is the discrimination of individual weed plants, the pixel size should be approximately 1–4 cm, which corresponds to flight altitudes of 27 to 105 m in the case of the still RGB camera and from 19 to 74 m in the case of the multispectral camera. However, when weed patch detection is aimed, the remote images could have a pixel size of 5 cm or even greater, which corresponds to a flight altitude higher than 100 m in both cameras.

**Figure 9:**
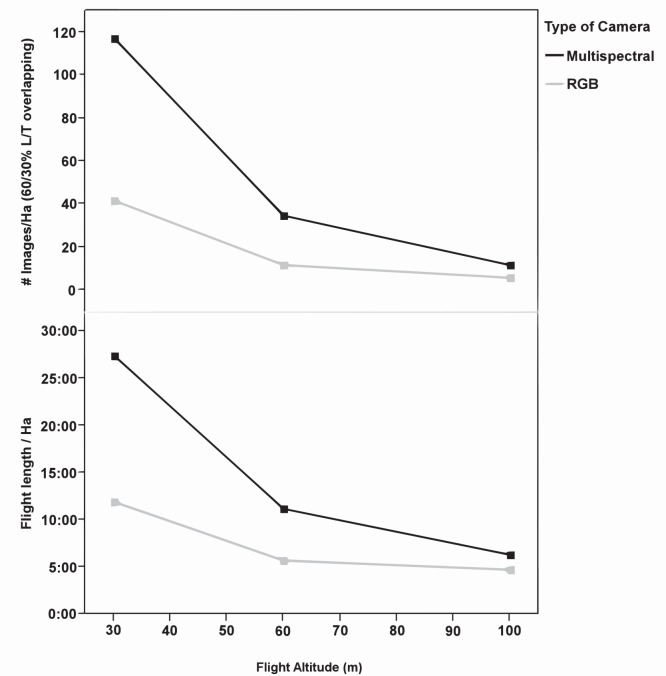
Flight length and number of images per ha as affected by flight altitude and camera.

The UAV acquired imagery with 60% forward lap and 30% side lap. From this overlapping and the camera sensor size, the WPE module calculated the number of images needed to capture the whole experimental field and, consequently, the time taken by the UAV to collect them at each flight altitude (Figure 9). The number of images per ha and the flight length were greater when using the multispectral camera, decreasing from 117 images ha^−1^ and 27 min at a 30 m altitude to 12 images ha^−1^ and 6 min at a 100 m altitude. For the still RGB camera, these variables ranged from 42 images ha^−1^ and 12 min at 30 m altitude to 6 images ha^−1^ 5 min at 100 m. A very large number of images can limit the mosaicking process because the number of images per hectare strongly increased at very low altitudes following an asymptotic curve. In addition, the operation timing is limited by the UAV battery duration. All these variables have strong implications in the configuration of the optimum flight mission for weed mapping in the early season, which involves two main conditions: 1) to provide remote images with a fine spatial resolution to guarantee weed discrimination, and 2) to minimise the operating time and the number of images to reduce the limitation of flight duration and image mosaicking, respectively.

### 3. Effect of Flight Altitude on Image Spectral Resolution

Spectral information captured by each camera at three flight altitudes was studied to determine significant differences at the pixel level between class 1 and class 2 in the two phases previously mentioned, i.e. between vegetation cover and bare soil, and between weeds and crop. The range and average spectral pixel values of the VIs, and M-statistics are shown in Table 3.

**Table 3:** Least Significant Differences (LSD) test at P≤0.01 and Spectral Separability according to the M-statistic between crop and weed plants and vegetation and bare soil as affected by vegetation index, type of camera and flight altitude.

			Vegetation *vs.* Bare soil discrimination										Crop *vs.* Weed discrimination									
			Vegetation				Bare soil				LSD test(Prob>F)	M (b)	Crop				Weed				LSD test(Prob>F)	M (b)
Flight altitude	Type of camera	VIs(a)	Max	Min	Mean	±SD	Max	Min	mean	±SD			Max	Min	mean	±SD	Max	Min	Mean	±SD		
30-m	RGB	NGRDI	0.11	−0.02	0.04	±0.03	−0.08	−0.11	−0.09	±0.01	<0.01	3.61	0.09	0.00	0.05	±0.02	0.11	−0.02	0.03	±0.03	0.10	0.32
		ExG	0.34	0.10	0.21	±0.06	0.02	−0.01	0.00	±0.01	<0.01	2.93	0.34	0.20	0.27	±0.04	0.20	0.10	0.16	±0.01	<0.01	1.61
	Multispectral	NDVI	0.73	0.45	0.58	±0.07	−0.15	−0.19	−0.16	±0.01	<0.01	8.90	0.73	0.52	0.61	±0.06	0.68	0.45	0.55	±0.07	0.03	0.42
		NGRDI	0.35	−0.04	0.14	±0.12	−0.20	−0.27	−0.23	±0.02	<0.01	2.75	0.35	0.15	0.24	±0.06	0.17	−0.04	0.05	±0.06	<0.01	1.59
		ExG	0.18	−0.01	0.08	±0.07	−0.05	−0.09	−0.06	±0.01	<0.01	1.94	0.18	0.11	0.15	±0.02	0.06	−0.01	0.02	±0.02	<0.01	3.02
60-m	RGB	NGRDI	0.06	−0.03	0.01	±0.02	−0.08	−0.10	−0.09	±0.01	<0.01	3.53	0.06	−0.03	0.01	±0.02	0.06	−0.02	0.01	±0.02	0.85	0.03
		ExG	0.26	0.11	0.18	±0.04	0.03	−0.01	0.01	±0.01	<0.01	3.50	0.26	0.14	0.20	±0.04	0.20	0.11	0.15	±0.03	<0.01	0.79
	Multispectral	NDVI	0.51	0.15	0.35	±0.09	−0.08	−0.11	−0.10	±0.01	<0.01	4.52	0.51	0.23	0.38	±0.09	0.46	0.15	0.33	±0.08	0.10	0.31
		NGRDI	0.23	−0.04	0.08	±0.07	−0.11	−0.13	−0.12	±0.01	<0.01	2.77	0.23	0.03	0.12	±0.06	0.11	−0.04	0.04	±0.04	<0.01	0.81
		ExG	0.28	0.10	0.18	±0.05	0.07	0.05	0.06	±0.01	<0.01	1.91	0.28	0.13	0.22	±0.04	0.19	0.10	0.14	±0.02	<0.01	1.19
100-m	RGB	NGRDI	0.04	−0.09	−0.02	±0.04	−0.09	−0.12	−0.10	±0.01	<0.01	1.67	0.04	−0.07	−0.01	±0.04	0.04	−0.09	−0.03	±0.03	0.13	0.28
		ExG	0.23	0.05	0.14	±0.05	0.02	−0.01	0.01	±0.01	<0.01	2.20	0.23	0.12	0.16	±0.05	0.22	0.05	0.12	±0.05	0.02	0.46
	Multispectral	NDVI	0.64	0.23	0.43	±0.10	−0.13	−0.16	−0.14	±0.01	<0.01	5.25	0.64	0.34	0.47	±0.09	0.56	0.23	0.39	±0.09	0.04	0.40
		NGRDI	0.20	−0.12	0.01	±0.09	−0.21	−0.24	−0.21	±0.01	<0.01	2.40	0.20	−0.05	0.08	±0.07	0.02	−0.12	−0.05	±0.04	<0.01	1.16
		ExG	0.23	0.07	0.14	±0.05	0.03	0.00	0.02	±0.01	<0.01	2.28	0.23	0.12	0.18	±0.03	0.12	0.07	0.10	±0.02	<0.01	1.90

(a) Vegetation indices: NGRDI = (G−R)/(G+R); ExG = 2g – r – b; NDVI = (NIR−R)/(NIR+R).

(b) M-statistic = (MEAN_class1_−MEAN_class2_)/(σ_class1_+ σ_class2_).

First of all, it was crucial to explore the spectral differences between vegetation and bare soil to identify the potential to perform the first step of our research scheme, such an approach should point out the significant variations in spectral data of both classes, indicating which set of VIs, cameras and altitudes were able for their discrimination. All the indices showed significant differences between vegetation and soil and, in most cases, M-statistics performed reasonably well exceeding 2, being NDVI the index that achieved the highest spectral separability at the three flight altitudes. This is due to NDVI emphasises the spectral response of the NIR band which characterises vegetation vigour and it is less sensitive to soil background effects than the other two indices. The magnitude of M-statistic, usually higher than 2.5 (excepting for ExG at 30 m and 60 altitudes and multispectral camera), offer satisfactory results for a high robustness of vegetation discrimination in all the scenarios. Kaufman and Remer (1994) [32] reported *M* values ranging from 1.5 to 0.5 for mapping dense vegetation in forests, whereas Smith *et al*. (2007) [33] obtained *M* values between 0.24 and 2.18 for mapping burned areas. According to our findings, the *M* achieved a much higher value (*M* = 8.9 for multispectral camera and NDVI index) suggesting robust separability of classes. NDVI could be the best index to perform the first phase of the proposed classification strategy, although NGRDI and ExG also showed an overall good capacity for distinguishing vegetal cover, which would be very relevant due to RGB camera is much cheaper and easier to use than the multispectral camera.

**Figure 10:**
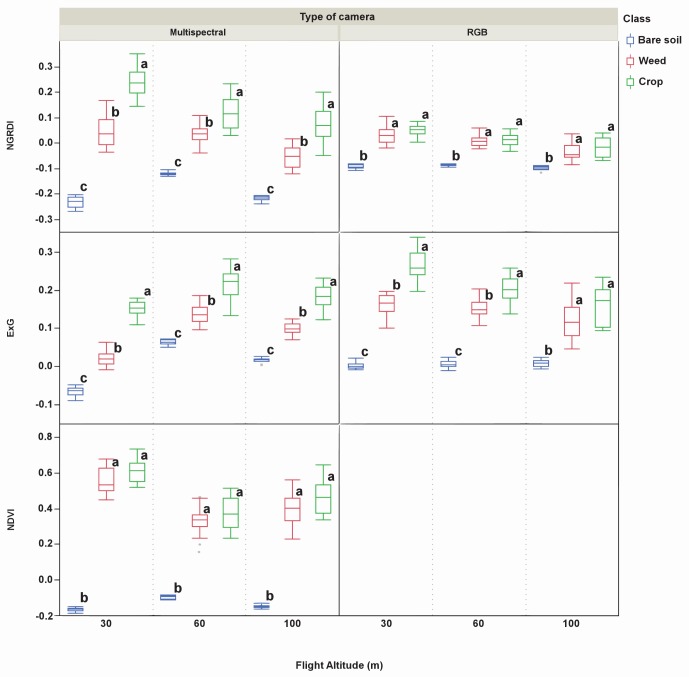
Vegetation index values of each class of soil cover (bare soil, weed and crop). The index values are affected by flight altitude and type of camera. Within a group, box-plots followed by the same letter do not differ significantly according to LSD test at P≤0.01.

In order to perform the second proposed phase, it is necessary to test if weeds and crop can be discriminated using either RGB camera or the multispectral sensor. As a general statement, the multispectral camera showed much higher capacity to discriminate crop and weeds than the RGB camera. The better performance of the multispectral camera may be caused by its narrow sensor bandwidth. This camera uses filters with a 10 nm bandwidth, which reduces the interferences caused by other wavelengths, while the RGB camera acquires information in three wider spectral wavebands from the entire visible spectrum. Thus, means of NGRDI and ExG were not significantly different for crop and weeds at any flight altitude and M-statistic values were the lowest ones, excepting for ExG at 30 m altitude where *M* = 1.61. However, even at this altitude, M-statistic value is quite lower than the obtained for ExG and the multispectral camera (*M* = 3.02). A preliminary conclusion could be that the RGB camera is able to discriminate weeds and crop using images from ExG at 30 m altitude. However, one of the key question to elucidate at this point is to determine if *M* = 1.61 provides enough robustness for mapping weeds and crop. That doubt could be clarified going to Figure 10 which shows the significant spectral differences among soil, weeds and crop in all the scenarios. Note that spectral differences among soil, and weeds and crop at 30 m altitude for ExG and RGB camera are clearly significant; however, the range of the standard deviation (see points in Fig. 10) of weeds and crop causes an overlapping which could produce a deficient discrimination between weeds and crop. Therefore, Table 3 offers an overall overview of separation between vegetation and soil, and weeds and crop; however these results must be deeply studied observing the ranges of minimum and maximum spectral values of every VI (Table 3) and ranges of standard deviation (Figure 10).

In the multispectral camera, NGRDI and ExG were significantly different for weeds and crop in all the flight altitudes tested. However, despite these significant differences observed and as stated before, the M-statistic and Figure 10 must be taken into account since both help to quantify the risk of misclassification due to the overlapping between value ranges of the vegetation indices studied. For instance, at 60 m altitude, NGRDI showed a significant spectral difference for weeds and crop; however M-statistic was lower than 1 (*M* = 0.81). This indicates that, apart from a significant spectral difference, a poor separation is expected between pixels from weeds and crop. This can be clearly appreciated in Figure 10 where the range of the standard deviation between weeds and crop involves an overlapping of values and this is the reason for which having a significant spectral discrimination this is not sufficient to achieve a satisfactory separability (*M* higher than 1).

The case of ExG is different since this vegetation index showed significant spectral differences and *M* values higher than 1 at any flight altitude, although *M* was only slightly superior than 1 (*M* = 1.19) at 60 m altitude. This points out that a good separation would be expected at 30 m and probably at 100 m; however, have the significant spectral differences and *M* = 1.19 obtained in Table 3 sufficient discriminatory power to properly separate crop and weeds at 60 m altitude?. Figure 10 again shows that this magnitude of *M* probably is not as much as required to successfully reach this objective due to the apparent overlapping of box-plots of weeds and crop and, consequently, a much more difficult separation would be expected at 60 m altitude. The only index studied using the NIR band was NDVI and it was not able to discriminate between crop and weeds at any flight altitude; in fact, NDVI showed the lowest M-statistic values among the indices calculated from the multispectral camera.

As mentioned in the previous section and according to the objective of minimising the operating time and the number of images taken to reduce the limitation of UAV flight duration and image mosaicking, the optimum flight mission may be to capture images at the highest altitude possible. However, the highest spectral differences and *M* values of pixels were obtained at the lowest altitudes, i.e., pixel-based methods may be unsuccessful in weeds and crop discrimination in seedling stages at altitudes higher than 30 m due to the spectral similarity among these vegetation classes. Currently, spectral limitations may be solved by implementing advanced algorithms such as the object-based image analysis (OBIA) methodology [35]. The OBIA methodology identifies spatially and spectrally homogenous units named *objects* created by grouping adjacent pixels according to a procedure known as segmentation. Afterwards, multiple features of localisation, texture, proximity and hierarchical relationships are used that drastically increase the success of image classification [36], [37]. In crop fields at an early stage, the relative position of the plants in the crop rows, rather than their spectral information, may be the key feature to distinguishing them. Consequently, every plant that is not located in the crop row can be assumed to be a weed. Therefore, according our results a strategy for a robust classification of UAV images could be developed involving two steps: 1) discriminating vegetation (weeds and crop) from bare soil by using spectral information, and 2) discriminating weeds from crop-rows using the OBIA methodology. Therefore, future investigations will be essential to determine the potential of OBIA techniques to distinguish and map weeds and crop using UAV imagery at higher flight altitudes and taken when weeds and crop are at the early phenological stages. Our recent research using OBIA methodology has shown the improvement of using satellite imagery for mapping crops [38] [37] or weeds at late phenological stages in winter wheat [12]. Our hypothesis for further work is based on the idea that the OBIA methodology has confirmed to be a powerful and flexible algorithm adaptable in a number of agricultural situations. The main aim would be to discriminate and map early weeds to enhance the decision making process for developing in-season ESSWM at high altitudes using RGB and ExG index compared to multispectral camera and the pixel-based image analysis. This would allow reducing the number of UAV imagery to improve the performance of the UAV (flight length and efficiency of energy supply) and the mosaicking process. This approach could be a more profitable method for mapping early weed infestations due to both, the covering of larger crop surface area and RGB cameras are cheaper and economically more affordable than multispectral cameras. Considering that the UAV development is a substantial investment, the possibility of using RGB cameras would reduce significantly the additional costs.

### Conclusions

Weeds are distributed in patches within crops and this spatial structure allows mapping infested-uninfested areas and herbicide treatments can be developed according to weed presence. The main objectives of this research were to deploy an UAV equipped with either, RBG or multispectral cameras, and to analyze the technical specifications and configuration of the UAV to generate images at different altitudes with the high spectral resolution required for the detection and location of weed seedlings in a sunflower field for further applications of ESSWM. Due to its flexibility and low flight altitude, the UAV showed ability to take ultra-high spatial resolution imagery and to operate on demand according to the flight mission planned.

The image spatial resolution, the area covered by each image and the flight timing varied according to the camera specifications and the flight altitude. The proper spatial resolution was defined according to each specific objective. A pixel lower than 4 cm was recommended to discriminate individual weed plants, which corresponded to flight altitudes below 100 m. If the objective was weed patch detection, the UAV can fly to a higher altitude to obtain remote images with pixels of 5 cm or greater. However, the number of images needed to cover the whole field could limit the flight mission at a lower altitude due to the increased flight length, problems with the energy supply, and the computational capacity of the mosaicking software.

Spectral differences between weeds, crop and bare soil were significant for NGRDI and ExG indices, mainly at a 30 m altitude. At higher altitudes, many weed and crop pixels had similar spectral values, which may increase discrimination errors. Greater spectral separability was obtained between vegetation and bare soil with the index NDVI, suggesting the employment of multispectral images for a more robust discrimination. In this case, the strategy for improving the image mosaicking and classification could be to implement the OBIA methodology to include features of localisation and proximity between weed and crop plants. An agreement among spectral and spatial resolutions is needed to optimise the flight mission according to the size of the smaller objects to be discriminated (weed plants or weed patches).

The information and results herein presented can help in the selection of an adequate sensor and to configure the flight mission for ESSWM in sunflower crops and other similar crop row scenarios (e.g., corn, sugar beet, tomato). Despite the initial complexity of management of the UAV and its components and software, and after a period of training the pilots and operators, the described workflow can be applied recursively.
